# NUSAP1 is Upregulated by Estrogen to Promote Lung Adenocarcinoma Growth and Serves as a Therapeutic Target

**DOI:** 10.7150/ijbs.100188

**Published:** 2024-10-07

**Authors:** Shaoping Zhang, Xiaozhen Zhang, Wenjian Huang, Ganling Jiang, Yuanxin Mo, Liuxia Wei, Pingming Fan, Maojian Chen, Wei Jiang

**Affiliations:** 1 Department of Respiratory Oncology, Guangxi Medical University Cancer Hospital, Nanning 530021, China.; 2 Department of Thoracic Surgery and Oncology, the First Affiliated Hospital of Guangzhou Medical University, State Key Laboratory of Respiratory Disease & National Clinical Research Center for Respiratory Disease, Guangzhou 510120, China.; 3 Department of Radiation Oncology, Guangxi Medical University Cancer Hospital, Nanning 530021, China.; 4 Department of Breast Surgery, the Sixth Affiliated Hospital of South China University of Technology, the Sixth Clinical College of South China University of Technology, Foshan 528225, China.; 5 Department of pharmacy, the Second Affiliated Hospital of Guangzhou Medical University, Guangzhou 510145, China.; 6 Department of Medical Oncology, Ruikang Hospital Affiliated to Guangxi University of Chinese Medicine, Nanning 530000, China.; 7 Department of Breast Surgery, the First Affiliated Hospital of Hainan Medical University, Haikou 570102, Hainan, China.; 8 Guangdong Provincial Key Laboratory of Malignant Tumor Epigenetics and Gene Regulation, Guangdong-Hong Kong Joint Laboratory for RNA Medicine, Sun Yat-Sen Memorial Hospital, Sun Yat-Sen University, Guangzhou 510120, China.

**Keywords:** nucleolar and spindle-associated protein 1, lung adenocarcinoma, estrogen, fulvestrant, entinostat

## Abstract

Nucleolar and spindle-associated protein 1 (NUSAP1), a microtubule-associated protein, has been recently identified to exhibit aberrant expression patterns that correlate with malignant tumorigenesis and progression across various cancer types. However, the specific regulatory mechanisms and potential targeting therapies of NUSAP1 in lung adenocarcinoma (LUAD) remain largely elusive. In this study, by conducting bioinformatics analyses as well as *in vitro* and* in vivo* experiments, we identified that NUSAP1 was significantly upregulated in LUAD, with a notable correlation with poorer overall survival, higher scores for immunogenicity and immune infiltration, as well as increased sensitivity to conventional chemotherapeutic drugs such as paclitaxel, docetaxel and vinorelbine in LUAD. Functionally, NUSAP1 overexpression significantly promoted LUAD cell proliferation, while its knockdown markedly suppressed this process. Interestingly, our results revealed that NUSAP1 upregulation was mediated by estrogen via ERβ activation. Furthermore, we identified entinostat as a novel inhibitor of NUSAP1. Pharmacological targeting ERβ/NUSAP1 axis with fulvestrant (ERβ antagonist) or entinostat (novel NUSAP1 inhibitor) significantly reduced LUAD growth both *in vitro* and *in vivo*, which may represent effective alternative therapeutic strategies for patients with LUAD.

## Introduction

Non-small cell lung cancer (NSCLC) represents one of the leading cause of cancer-related mortality globally^1^. Among the histological types of NSCLC, lung adenocarcinoma (LUAD) comprises approximately 60% of newly diagnosed cases, exhibiting a substantial increase^1^, ^2^. Over the past decade, the identification of driver oncogenes, such as EGFR, ALK, ROS1, has facilitated the development of targeted therapeutic drugs, thereby greatly improving therapeutic outcomes for LUAD patients harboring these mutations. However, for patients without active mutations, the benefit of targeted therapy is limited, and immune checkpoint inhibitors (ICIs) or combinations with chemotherapy are the standard of care^3^-^5^. Notably, the efficacy of immunotherapy or combinations with chemotherapy remains suboptimal, highlighting the pressing need to identify novel effective therapeutic targets to develop alternative therapeutics to improve outcomes for LUAD patients.

Nucleolar and spindle-associated protein 1 (NUSAP1) is a microtubule-associated protein residing on chromosome 15q15.1^6^. NUSAP1 has been identified as a crucial mitotic regulator essential for multiple stages of cell division, including spindle assembly, chromosome segregation, cytokinesis, microtubule crosslinking, bundling, and attachment to chromosomes^6^-^8^. Notably, numerous studies have documented aberrant NUSAP1 overexpression as a key factor in tumor progression across diverse human cancer types, including gastric cancer^9^, bladder cancer^10^, and renal cell carcinoma^11^. For instance, NUSAP1 plays a critical role in metastasis of pancreatic ductal adenocarcinoma by regulating lactate dehydrogenase A-mediated glycolysis^12^. In prostate cancer, NUSAP1 can be upregulated by E2F1 activation or loss of RB1 and thus promoting cancer invasion and metastasis^13^, ^14^. Moreover, NUSAP1 sustains cancer stemness to promote early recurrence of hepatocellular carcinoma^15^. Intriguingly, study has reported that NUSAP1 may influence immune responses through its interaction with DNA or RNA and implicated in the proliferation of acute myeloid leukemia (AML) cells 16. Besides, NUSAP1 has been reported to be correlated with immune infiltration in cancers including thyroid carcinoma^17^, breast cancer^18^and bladder cancer^19^. Recently, there is a study reported that aberrant NUSAP1 is closely involved in the progression and poor prognosis of NSCLC^20^. Despite these advances, the precise role and underlying regulatory mechanisms of NUSAP1 in LUAD remain largely elusive. Therefore, a thorough investigation of the specific role and regulatory mechanisms of NUSAP1 in LUAD may hold great prospects in developing new therapeutic strategy for patients with LUAD.

Estrogens, including estradiol (E2), estrone and estriol, act through their receptors, playing a crucial role in diverse biological functions, particularly among females^21^-^23^. Accumulating evidence has highlighted the important role of estrogen and its receptors in the tumorigenesis and development of LUAD^24^-^28^. For example, Huang *et al.* reported that E2 upregulates IL6 expression through the ERβ pathway to promote LUAD progression 28. Chen *et al.* demonstrated that E2 exerts a dual effect in lung cancer, affecting cancer cells and modulating the tumor-associated microenvironment, ultimately contributing to a poorer prognosis^29^. Notably, ERβ, a crucial estrogen receptor, emerges as a pivotal factor in tumor progression^30^-^32^, exhibiting high expression in NSCLC^33^. Remarkably, ERβ also plays a significant role in promoting osimertinib resistance in NSCLC^34^. In clinical observations, a conspicuously higher incidence of LUAD is observed among female patients, coupled with a higher prevalence of specific driver gene mutations^35^, ^36^. Recent investigations have revealed estrogen signaling as a crucial pathway activated in never-smoker LUAD patients without EGFR and ALK alterations^37^. All these findings point to a potential role of estrogen signaling in LUAD development. However, the precise underlying mechanism remains elusive and merits further investigation.

Here, we found that NUSAP1 expression was significantly upregulated in LUAD, exhibiting a significant correlation with poorer overall survival, higher scores for tumor mutation burden (TMB), neoantigen loads (NALs), immune infiltration, as well as higher expression of immune checkpoint blockade (ICB)-relevant genes. In addition, high NUSAP1 expression was associated with increased sensitivity to conventional chemotherapeutic drugs such as vinorelbine, docetaxel and paclitaxel, as well as the combined chemotherapy of carboplatin and paclitaxel in LUAD. Functionally, NUSAP1 overexpression significantly promoted LUAD cell proliferation, while its knockdown significantly suppressed this process. Interestingly, our results revealed that NUSAP1 upregulation was mediated by estrogen via ERβ activation, and treatment with fulvestrant, an ERβ antagonist, significantly reduced LUAD growth both *in vitro* and *in vivo* by targeting ERβ/NUSAP1 axis. Furthermore, entinostat was identified as a novel inhibitor of NUSAP1, exhibiting potent anti-LUAD growth effects in preclinical models. These findings indicate that NUSAP1 is a promising therapeutic target in LUAD, and pharmacological inhibition of NUSAP1 with fulvestrant or entinostat may offer novel therapeutic strategies for this malignancy.

## Materials and Methods

### Public data access

RNA sequencing data, along with clinical details pertaining to LUAD cases, were retrieved from the Cancer Genome Atlas (TCGA) database (^https://cancergenome.nih.gov^). Additionally, RNA sequencing matrices of normal lung tissues were downloaded from Genotype Tissue Expression Project (GTEx) database (^https://commonfund.nih.gov/GTex^). Furthermore, gene expression matrices were extracted from the Gene Expression Omnibus (GEO) database (^https://www.ncbi.nlm.nih.gov/gds^). R software was utilized for the integration, analysis and visualization of the obtained data. The additional renowned web-based databases employed in this study were described below.

### Differential expression analysis

The differential expression of NUSAP1 mRNA between normal lung tissues and LUAD tissues was analyzed using data obtained from databases of GTEx, TCGA, GEO (datasets: GSE43458, GSE31210, GSE30219, GSE75037, GSE32863), as well as the UALCAN^38^, ^39^ website database (^https://ualcan.path.uab.edu/^). Additionally, the differential expression of NUASP1 protein level was investigated based on data collected from the Clinical Proteomic Tumor Analysis Consortium (CPTAC)^40^ (^https://gdc.cancer.gov/^) and the Human Protein Atlas (HPA) 41 databases (^https://www.proteinatlas.org/^). Besides, in order to explore the effect of E2 and fulvestrant on the expression of NUSAP1, GEO datasets (GSE46924, GSE4668 (E2)); GSE2253 (fulvestrant)) were downloaded for analysis.

### Survival prognosis analysis

LUAD patients from the TCGA-LUAD cohort were divided into high and low groups based on the median expression level of NUSAP1. The Kaplan-Meier method was then used to analyze overall survival (OS) of these patients. Besides, GEO datasets (GSE31210, GSE30219, GSE33745, GSE50081, GSE68465) were utilized to further verify the influence of NUSAP1 on OS of LUAD patients.

### Gene mutation analysis

The TIMER2.0^42^ platform (^http://timer.cistrome.org/^) was utilized to investigate the association between the somatic mutations of common driver genes in LUAD and NUSAP1 expression. The muTarget website database^43^ (^https://www.mutarget.com^) was employed to identify other genes whose somatic mutations may relate to NUSAP1 overexpression in LUAD, with the mutation prevalence threshold setting at a minimum of 3%.

### Functional Analysis

The CancerSEA^44^ website database (^http://biocc.hrbmu.edu.cn/CancerSEA/home.jsp^) was utilized to examine the expression of NUSAP1 across 14 functional states in LUAD at the single-cell level. In addition, differentially expressed genes (DEGs) were identified from the TCGA-LUAD cohorts between high- and low-NUSAP1 groups, with a threshold of log_2_ (fold change) > 1.5 and adjust *p* < 0.05. Gene Ontology (GO) and Kyoto Encyclopedia of Genes and Genomes (KEGG) functional analyses were performed to annotate the DEGs related to NUSAP1.

### Immunogenicity, immune infiltration and ICB response analysis

The CAMOIP^45^ (^http://www.camoip.net/^) online tool was utilized to explore the influence of NUSAP1 expression on immunogenicity, immune infiltration and immune checkpoint blockade (ICB)-relevant genes expression in the TCGA-LUAD cohort. Indicators of immunogenicity encompass tumor mutation burden (TMB), neoantigen loads (NALs) and MANTIS score^46^. The fraction of 22 types of infiltrating immune cells were determined using the CIBERSORT algorithm^47^. The ICB-relevant genes examined comprised PDCD1, CD274, PDCD1LG2, IDO1, LAG3, TNFSF8, CTLA4, ICOS and HAVCR2.

### Drug sensitivity analysis

The Genomics of Drug Sensibility in Cancer (GDSC)^48^ website database (^https://www.cancerrxgene.org/^) was utilized to explore the influence of NUSAP1 on the half-maximal inhibitory concentration (IC50) of commonly administered chemotherapeutic and targeted therapeutic drugs for LUAD treatment. In addition, the Cancer Treatment Response gene signature DataBase (CTR-DB)^49^ (^http://ctrdb.cloudna.cn/home^) was employed to explore the response predictive value of NUSAP1 in combination therapeutic strategies for LUAD.

### Cell culture

Human lung adenocarcinoma cell lines (A549 and H1975) and human embryo kidney (HEK) 293T cell line were purchased from American Type Culture Collection (ATCC; Manassas, VA, USA). A549 and H1975 cells were cultured in Roswell Park Memorial Institute (RPMI) 1640 medium (Gibco; Rockville, MD, USA) without phenol red. HEK293T cells were maintained in Dulbecco's modified Eagle's medium (DMEM). Cell culture mediums were supplemented with 10% fetal bovine serum (Gibco; Rockville, MD, USA), 100 µg/mL streptomycin (Hyclone; Logan, UT, USA), and 100 U/mL penicillin (Hyclone; Logan, UT, USA). All cells were cultured in a humidified incubator at 37 °C and 5% CO_2_. All cell lines were routinely detected to ensure negative mycoplasma contamination.

### Plasmid construction and lentivirus-mediated transfection

The cDNAs were obtained by PCR amplification and the CDS region of human NUSAP1 or ERβ were cloned into a pSin vector. For lentiviral particle production, lenti-viral plasmids pSin-NUSAP1 or pSin-ERβ were co-transfected with psPAX2 and pMD2G into HEK293T cells using Lipofectamine™ 3000 (Invitrogen; MA, USA) according to the manufacturers' protocols. Following 48 h transfection, the supernatant containing viruses were harvested, filtered, and used for infecting target cells (A549 and H1975) in the presence of 8 mg/mL polybrene (Sigma-Aldrich; St. Louis, MO, USA). Finally, the transduced cells were selected by treatment with 2 μg/mL puromycin to generate cells with NUSAP1 or ERβ stable overexpression.

### siRNAs and transfection

The small interfering RNA (siRNA) sequences were synthesized by Guangzhou IGE Biotechnology Ltd. (Guangzhou, China). The siRNA constructs were as follows: siRNA-NUSAP1(siNUSAP1): 5'-GUCAACAGAAUUAACUUCUdTdT-3'(sense) and 5'-AGAAGUUAAUUCUGUUGACdTdT-3'(anti-sense). siRNA-ERβ (siERβ): 5'-GCAAAGAGGGCUCCCAGAAdTdT-3'(sense), 5'-UUCUGGGAGCCCUCUUUGCdTdT-3'(anti-sense). siRNA-negative control (siNC): 5'-UUCUCCGAACGUGUCACGUdTdT-3' (sense) and 5'-ACGUGACACGUUCGGAGAAdTdT-3' (anti-sense). A549 and H1975 cells were transfected with siRNAs using Lipofectamine™ 3000 (Invitrogen; MA, USA) according to the manufacturers' protocols. The efficiency of NUSAP1 knockdown was assessed by western blot.

### CCK8 assay

The indicated cells were seeded in 96-well plates at a concentration of 3×10^3^ cells/100μL/well. Six wells were repeated in each group. After overnight incubation to attachment, cells were treated with indicated concentrations of E2, fulvestrant or entinostat for 0 h, 24 h, 48 h, 72 h and 96 h, respectively. Then each well was added 10 μL CCK8 solution (Dojindo Laboratories; Kumamoto, Japan) and incubated for 2 h. The absorbance at 450 nm was measured by enzyme immunoassay instrument.

### Colony formation assay

The indicated cells were plated into 12-well plates (500 cells/well). After 7 days, the visible cell colonies were fixed with 4% paraformaldehyde for 15 min and then stained with 1% crystal violet for 20 min. Finally, the cell colonies (≥ 50 cells) were photographed and counted.

### Western blot analysis

Western blot analysis was preformed using a slightly modified protocol of a previously described method^50^. Briefly, cells were lysed on ice for half an hour in RIPA lysis buffer (Beyotime Biotechnology Co., Ltd.; Shanghai, China) containing protease and phosphatase inhibitor cocktail (Beyotime Biotechnology Co., Ltd.; Shanghai, China). BCA protein assay kit (Beyotime Biotechnology Co., Ltd.; Shanghai, China) was utilized for protein quantification. An aliquot of protein was seperated by 10% SDS-PAGE, and transferred onto polyvinylidene difluoride (PVDF) membranes. After blocking with QuickBlock™ Western Blot Blocking Buffer (Beyotime Biotechnology Co., Ltd.; Shanghai, China) at room temperature for half an hour, blots were incubated at 4 °C overnight with the following specific antibodies: anti-NUSAP1(1:5000 dilution; Cat No. 12024-1-AP), anti-ERβ (1:2000 dilution; Cat No. 14007-1-AP) or GAPDH (1:5000 dilution; Cat No. 10494-1-AP) (All from Proteintech Group, Inc; Wuhan, China). After washing three times with TBST, blots were incubated with the corresponding HRP-conjugated secondary antibody (Beyotime Biotechnology Co., Ltd.; Shanghai, China) at room temperature for an hour, and then washed with TBST three times. The immunoreactivity was determined using a BeyoECL Moon kit (Beyotime Biotechnology Co., Ltd.; Shanghai, China). The signal intensity was visualized using an enhanced chemiluminescence detection system (BioRad; Hercules, CA, USA).

### Molecular docking

The 2D structures of the small molecular compounds listed in** Figure 11A** were downloaded from the PubChem website (^https://pubchem.ncbi.nlm.nih.gov/^) and translated into 3D structures with energies minimized using Chem3D 17.1. software. The structure of NUASP1 protein was downloaded from the Protein Data Bank database^51^ (^https://www.rcsb.org/^) and imported into AutoDockTools 1.5.7 for removal of water molecules, hydrogenation, and charge addition. After setting the grid parameters, AutoDock Vina (^https://vina.scripps.edu/^) was used to execute molecular docking. PyMOL and Discovery Studio 2019 softwares were utilized to visualize the docking results.

### Animal studies

Female BALB/c nude mice (5 weeks of age, 18-20 g) were obtained from Guangxi Medical University Experimental Animal Center and maintained under specific pathogen-free condition. All mice procedures were approved by the Guangxi Medical University Committee on Use and Care of Animals.

Xenograft mouse model for E2 and fulvestrant treatment, mice firstly received ovariectomy under isoflurane inhalation anesthesia to avoid the influence of endogenous estrogen. Then A549 cells (5 × 10^6^) were subcutaneously injected into the right flank of each mouse. Once the tumor volume reached approximately 100 mm^3^, mice were randomly divided into four groups (five mice per group): vehicle (saline), E2 (0.09 mg/kg, i.h, q3d), fulvestrant (2.4 mg/kg, i.h, q3d), and E2 plus fulvestrant groups. E2 (Cat No. HY-B0141) and fulvestrant (Cat No. HY-13636) were purchased from MedChemExpress (Monmouth Junction, NJ, USA). The doses of E2 and fulvestrant were set referring to the previous studies^27^, ^52^.

Xenograft mouse model for entinostat treatment, A549 cells (5 × 10^6^) stably expressing either vector or NUSAP1 were subcutaneously injected into the right flank of each mouse (ten mice in each setting). Once the tumor volume reached approximately 100 mm^3^, two settings of mice were further divided into four groups (five mice in each group): Group I (Control group) and Group II (OE-NUSAP1 group) received an equal volume of saline (1% tween 80) for intraperitoneal administration; Group Ⅲ (entinostat group) and Group Ⅳ (OE-NUSAP1 plus entinostat group) received an intraperitoneal injection of entinostat (20 mg/kg, q3d) (Cat No. HY-12163; MedChemExpress). The dosages of entinostat were as previously described^53^.

The mouse body weight and tumor growth were monitored every three days. Tumor volume (V) was estimated using the formula: V = L(length) × W (width)^2^/2. At the end of the experiments, the mice were euthanized, and the tumors were dissected and evaluated.

### Statistical analysis

Statistical analysis was conducted using R software (Version 4.1.1) or GraphPad Prism 8 Software (San Diego, CA, USA). For public data analysis, the Wilcoxon rank-sum test was used to compare the gene expression between two groups. Significance of survival analysis was calculated using Kaplan-Meier analysis with the Log-rank test. Measurement data of* in vitro* and *in vivo* experiments were presented as means ± standard deviation (SD) and analyzed using Student's t-test for two groups or one-way analysis of variance (ANOVA) for multiple groups. *p* < 0.05 was considered statistically significant.

## Results

### NUSAP1 is highly expressed in LUAD

**Figure 1:**
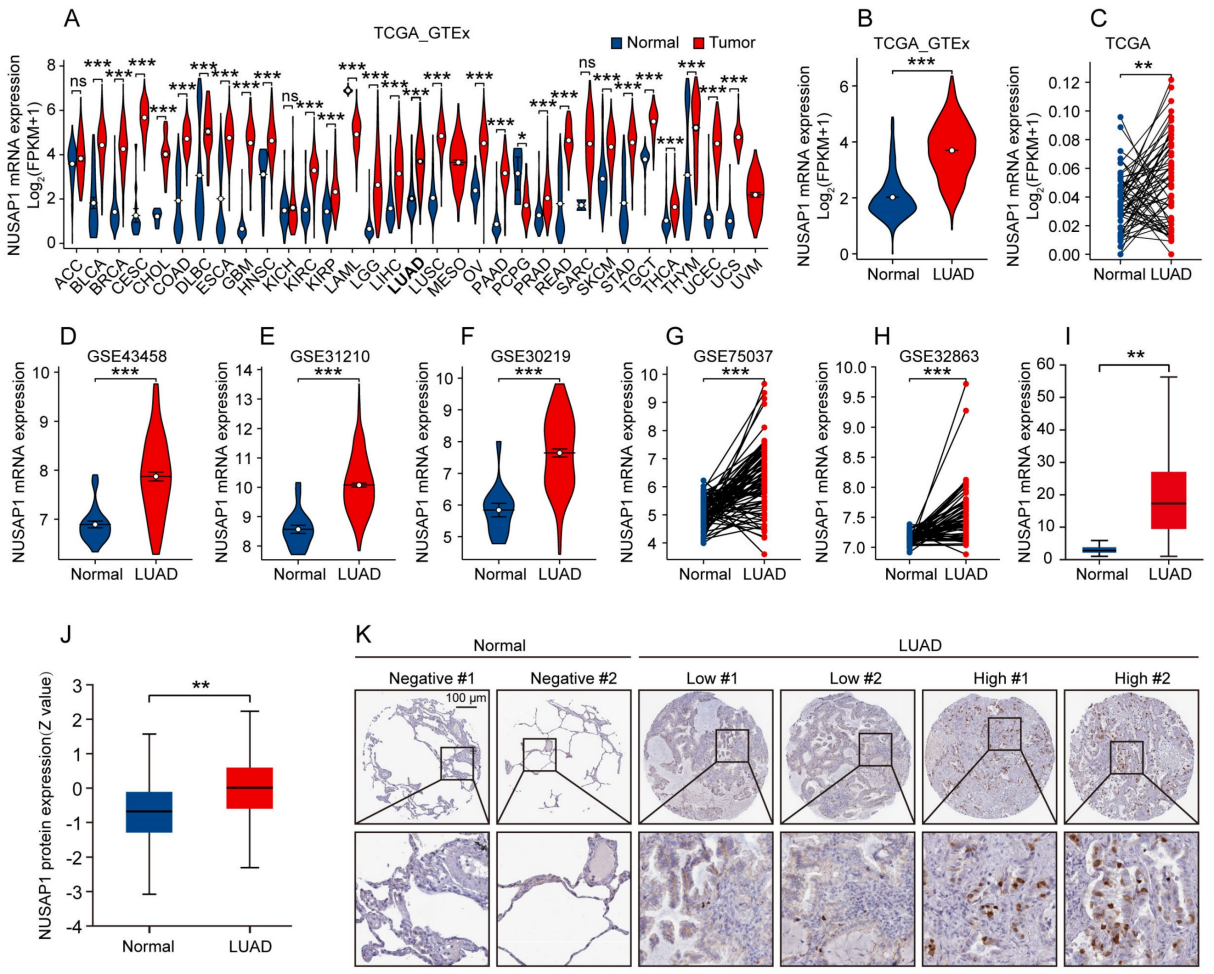
** NUSAP1 is highly expressed in LUAD**. **(A)** NUSAP1 mRNA expression in pan-cancer including LUAD and corresponding organ normal tissues from TCGA & GTEx databases. (^*^p < 0.05; ^***^p < 0.001; ns, not significant). **(B)** NUSAP1 mRNA expression in LUAD and unpaired normal lung tissues from TCGA & GTEx databases. (^***^p < 0.001). **(C)** NUSAP1 mRNA expression in LUAD and paired normal lung tissues from TCGA database. (^**^p < 0.01). **(D-F)** NUSAP1 mRNA expression in LUAD and unpaired normal lung tissues from independent GEO datasets: GSE43458 **(D)**, GSE31210 **(E)** and GSE30219 **(F)**. (^***^p < 0.001). **(G-H)** NUSAP1 mRNA expression in LUAD and paired normal lung tissues from independent GEO datasets: GSE75037 **(G)** and GSE32863 **(H)**. (^***^p < 0.001). **(I)** NUSAP1 mRNA expression in LUAD and unpaired normal lung tissues from UALCAN website database. (^**^p < 0.01). **(J)** NUSAP1 protein expression in LUAD and normal lung tissues from CPTAC database. (^**^p < 0.01). **(K)** Representative immunohistochemical images of NUSAP1 protein staining in LUAD and normal lung tissues from Human Protein Atlas database.

Firstly, we assessed the mRNA expression of NUSAP1 across pan-cancer by analyzing data from the TCGA and GTEx database. Our results showed that, in comparison to normal samples, the expression levels of NUSAP1 mRNA were significantly elevated in most human cancer tissues, including LUAD (**Figure 1 A, B**). Consistent with these observations, a comparison between LUAD and paired normal lung tissues also showed a significant upregulation of NUSAP1 mRNA expression in LUAD (**Figure 1 C**). In addition, results from five independent GEO datasets (GSE43458, GSE31210, GSE30219, GSE75037, GSE32863) and UALCAN database reconfirmed the higher expression of NUSAP1 mRNA in LUAD compared to unpaired or paired normal lung tissues (**Figure 1 D-I**).

Subsequently, we further analyzed the protein expression of NUSAP1 in LUAD utilizing the CPTAC database. Our results showed a significantly higher expression of NUSAP1 protein in LUAD tissues compared to normal tissues (**Figure 1 J**). Likewise, data from the Human Protein Atlas database further corroborated our findings as evidenced by stronger NUSAP1 protein staining in LUAD tissues compared to normal lung tissues (**Figure 1 K**).

Collectively, these results indicate an abnormal expression of NUSAP1 in LUAD, suggesting its potential involvement in the development of LUAD.

### Prognostic potential of NUSAP1 in LUAD

**Figure 2:**
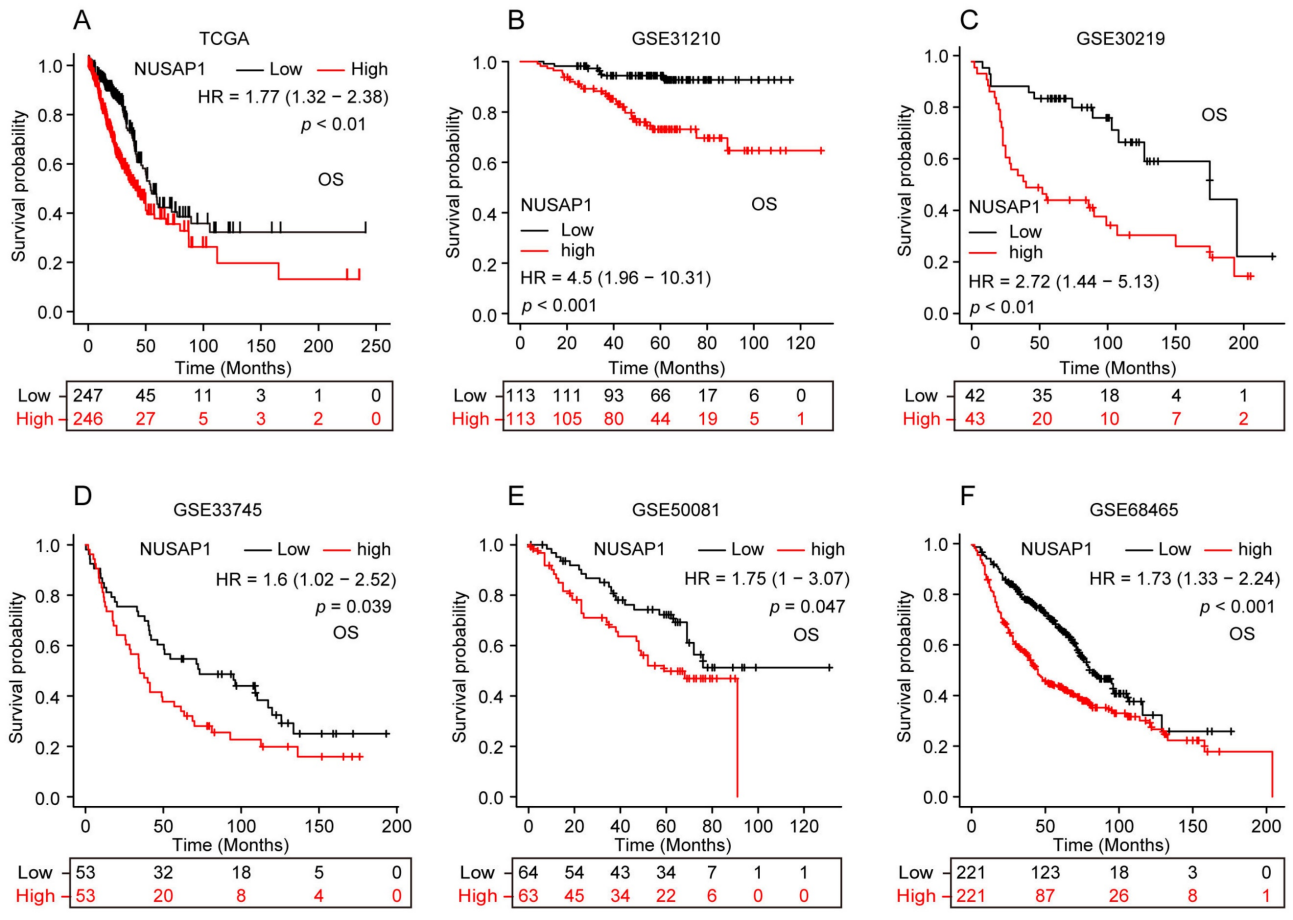
**Prognostic potential of NUSAP1 in LUAD patients. (A)** Kaplan-Meier curve of overall survival (OS) for LUAD patients with high- and low-NUSAP1 expression from TCGA-LUAD cohort. **(B-F)** Kaplan-Meier curves of OS for LUAD patients with high- and low-NUSAP1 expression from independent GEO datasets: GSE31210 **(B)**, GSE30219 **(C)**, GSE33745 **(D)**, GSE50081 **(E)** and GSE68465 **(F)**.

To further explore the potential association between NUSAP1 expression and prognosis of LUAD patients, we classified patients in TCGA-LUAD cohort into high- and low-NUSAP1 expression groups (cutoff: median) to perform survival analysis. The Kaplan-Meier survival analysis showed that patients with higher NUSAP1 expression had worse overall survival (OS) (**Figure 2 A**). Besides, analysis from five independent GEO datasets (GSE31210, GSE30219, GSE33745, GSE50081 and GSE68465) concurred with this observation (**Figure 2 B-F**), suggesting that NUSAP1 is closely positively associated with poor OS of LUAD patients.

### Correlation between NUSAP1 expression and LUAD driver genes mutation

**Figure 3:**
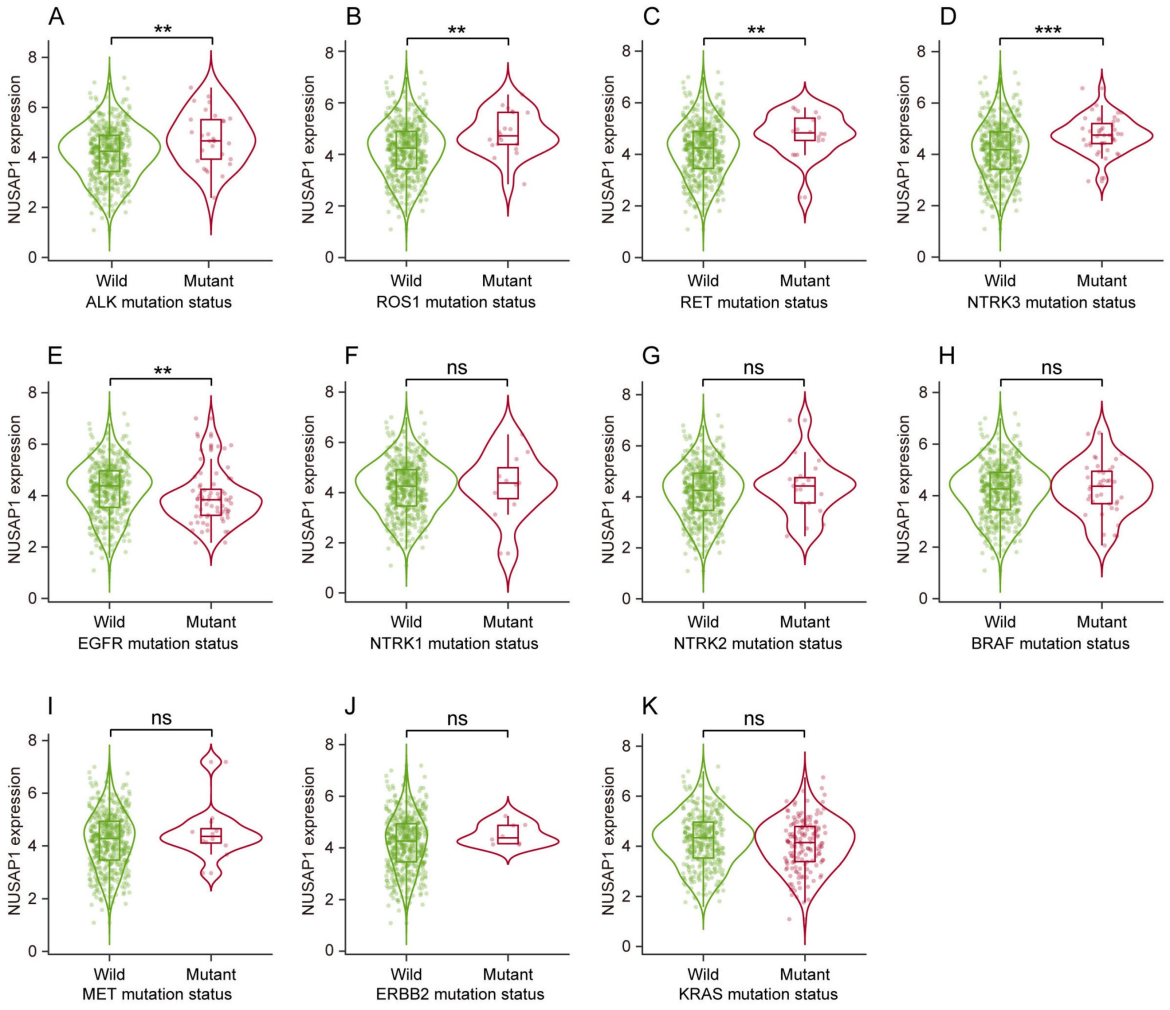
** Relationship between genetic abnormalities of LUAD driver genes and NUSAP1 expression from TIMER 2.0 website database. (A-K)** NUSAP1 expression in wild type and mutated groups of common LUAD driver genes, including ALK **(A)**, ROS1 **(B)**, RET **(C)**, NTRK3** (D)**, EGFR** (E)**, NTRK1** (F)**, NTRK2 **(G)**, BRAF** (H)**, MET **(I)**, ERBB2 **(J)**, KRAS **(K)**. (^**^p < 0.01; ^***^p < 0.001; ns, not significant).

Next, we sought to address whether NUSAP1 expression correlated with genetic abnormalities of LUAD driver genes (including EGFR, ALK, ROS1, BRAF, KRAS, MET, RET, ERBB2 and NTRK1/2/3), which are crucial for LUAD occurrence and progression. The results showed that, compare to the wild-type group, NUSAP1 was significantly overexpressed in mutated groups of ALK, ROS1, RET and NTRK3 (**Figure 3 A-D**) while inversely low-expressed in EGFR-mutated group (**Figure 3 E**). No significant NUSAP1 expression changes were observed between wild-type and mutated groups of NTRK1, NTRK2, BRAF, MET, ERBB2 and KRAS (**Figure 3 F-K**). Considering the current evidence primarily revealing a correlation between NUSAP1 overexpression and mutations of major driver genes including ALK, ROS1, RET and NTRK3 in LUAD, we speculate that NUSAP1 may involve in the tumorigenesis and progression of LUAD.

### Somatic mutations of genes showed altered NUSAP1 expression in LUAD

**Figure 4:**
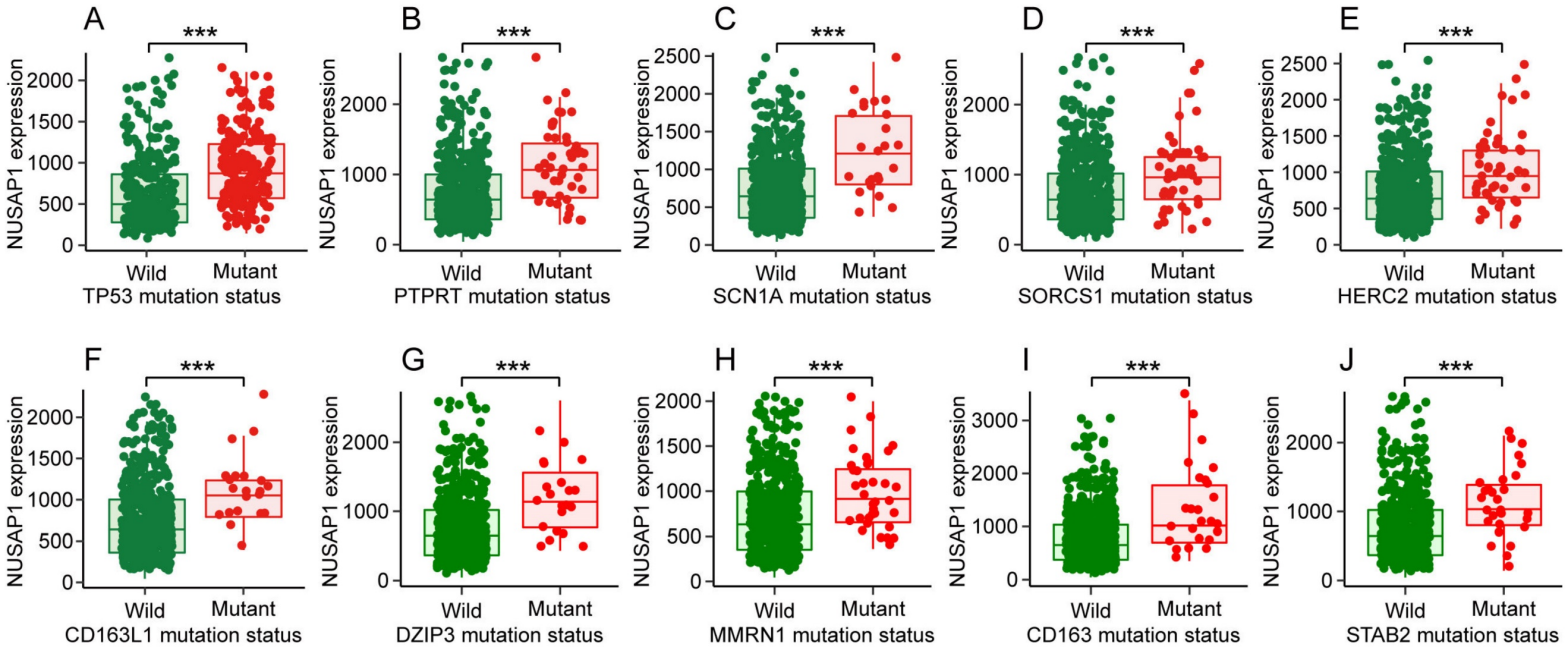
** Somatic mutations of the top 10 genes that showed altered NUSAP1 expression in LUAD from muTarget website database. (A-J)** NUSAP1 expression in wild type and somatic mutated groups of the top 10 genes that showed altered NUSAP1 expression in LUAD, including TP53** (A)**, PTPRT **(B)**, SCN1A **(C)**, SORCS1** (D)**, HERC2 **(E)**, CD163L1** (F)**, DZIP3 **(G)**, MMRN1** (H)**, CD163** (I)**, STAB2 **(J)**. (^***^p < 0.001).

By analyzing the muTarget database, we further discovered that somatic mutations in various genes showed altered expression of NUSAP1 in LUAD, such as the top ten genes of TP53, PTPRT, SCN1A, SORCS1, HERC2, CD163L1, DZIP3, MMRN1, CD163 and STAB2 (**Figure 4 A-J**). These findings suggest the significant role of NUSAP1 in the development of LUAD.

### NUSAP1-related functional analyses in LUAD

**Figure 5:**
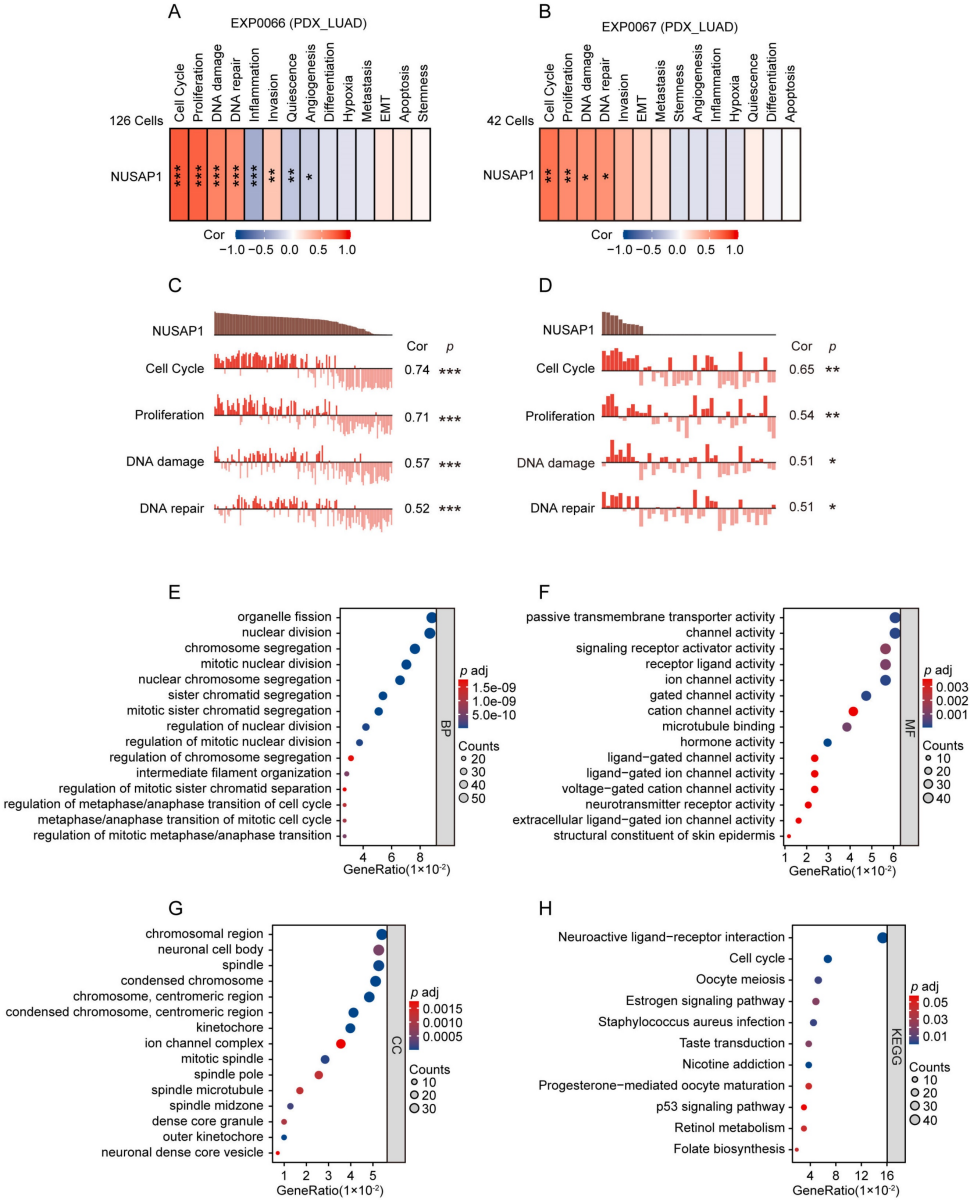
** NUSAP1-related functional analyses in LUAD. (A-B)** Relationship between NUSAP1 expression and 14 different cancer functional states in LUAD at single cell level base on two datasets (EXP0066 and EXP0067) from CancerSEA website database (^*^p < 0.05; ^**^p < 0.01; ^***^p < 0.001).** (C-D)** Association between NUSAP1 expression and the top 4 significant functional states in LUAD at single cell level base on two datasets (EXP0066 and EXP0067) from CancerSEA website database. (^*^p < 0.05; ^**^p < 0.01; ^***^p < 0.001). **(E-G)** GO terms functional analyses of NUSAP1-related upregulated differentially expressed genes (DEGs) from TCGA-LUAD cohort. BP, biological process **(E)**, CC, cellular component **(F)**, MF, molecular function **(G)**. **(H)** KEGG pathway analyses of NUSAP1-related upregulated DEGs from TCGA-LUAD cohort.

To further validate the functional role of NUSAP1 in LUAD, we employed two scRNA sequencing datasets (EXP0066 and EXP0067) in CancerSEA database to analyze the functional states associated with NUSAP1 expression at the single-cell level. The results showed that in both datasets, NUSAP1 expression positively correlated with functional states pertinent to cell cycle, proliferation, DNA damage and DNA repair in LUAD (**Figure 5 A-D**). Moreover, GO and KEGG analyses showed that, the NUSAP1-related upregulated DEGs from TCGA-LUAD cohort enriched mainly in terms involving in the regulation of cell mitotic division and cell cycle (**Figure 5 E-H**). These results verify the pivotal role of NUSAP1 in the malignant process of LUAD.

### Analysis between NUSAP1 and immunogenicity, immune infiltration or ICB-relevant genes expression in LUAD

**Figure 6:**
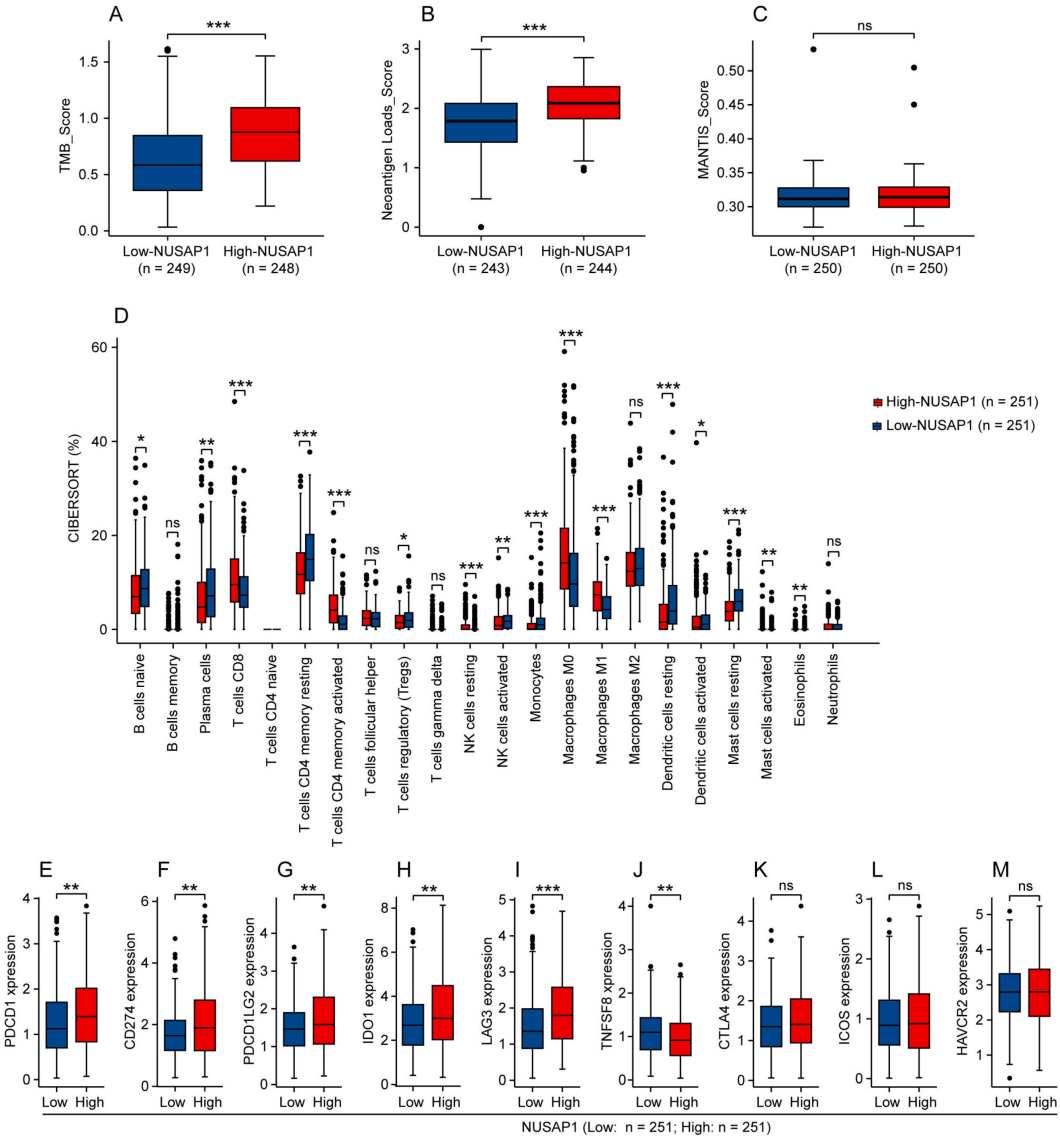
** Analysis between NUSAP1 and immunogenicity, immune infiltration or ICB-relevant genes expression in LUAD. (A-C)** Immunogenicity indicators of tumor mutation burden (TMB)** (A)**, neoantigen loads (NALs) **(B)** and MANTIS **(C)** score in high- and low-NUSAP1 expression groups from TCGA-LUAD cohort. **(D)** The fraction of 22 types of infiltrating immune cells in high- and low-NUSAP1 expression groups from TCGA-LUAD cohort. **(E-M)** Expression of classical ICB-relevant genes including PDCD1 **(E)**, CD274 **(F)**, PDCD1LG2 **(G)**, IDO1 **(H)**, LAG3** (I)**, TNFSF8** (J)**, CTLA4 **(K)**, ICOS **(L)** and HAVCR2 **(M)** in high- and low-NUSAP1 expression groups from TCGA-LUAD cohort.

We subsequently investigated whether NUSAP1 is associated with immunogenicity, immune infiltration and ICB-relevant genes expression in LUAD. By analyzing the TCGA-LUAD cohort utilizing the CAMOIP online tool, we found that high-NUSAP1 expression group exhibited higher scores for tumor mutation burden (TMB) and neoantigen loads (NALs), but not MANTIS (**Figure 6 A-C**). Furthermore, analysis based on the CIBERSORT algorithm showed that LUAD patients with high NUSAP1 expression were infiltrated with greater proportion of immune cells, such as CD8^+^ T cells, activated CD4^+^ memory T cells, M0 and M1 macrophages (**Figure 6 D**). Concurrently, we found that elevated NUSAP1 expression was associated with increased expression of ICB-relevant genes, including PDCD1, CD274, PDCD1LG2, IDO1 and LAG3, and inversely decreased TNFSF8 expression (**Figure 6 E-J**). No significant expression changes of CTLA4, ICOS and HAVCR2 were observed between high- and low-NUSAP1 expression groups (**Figure 6 K-M**). These findings suggest a significant association between NUSAP1 and immune microenvironment in LUAD, which may influence the response to immunotherapy.

### Different chemotherapeutic and targeted therapeutic responses based on NUSAP1 expression in LUAD

**Figure 7:**
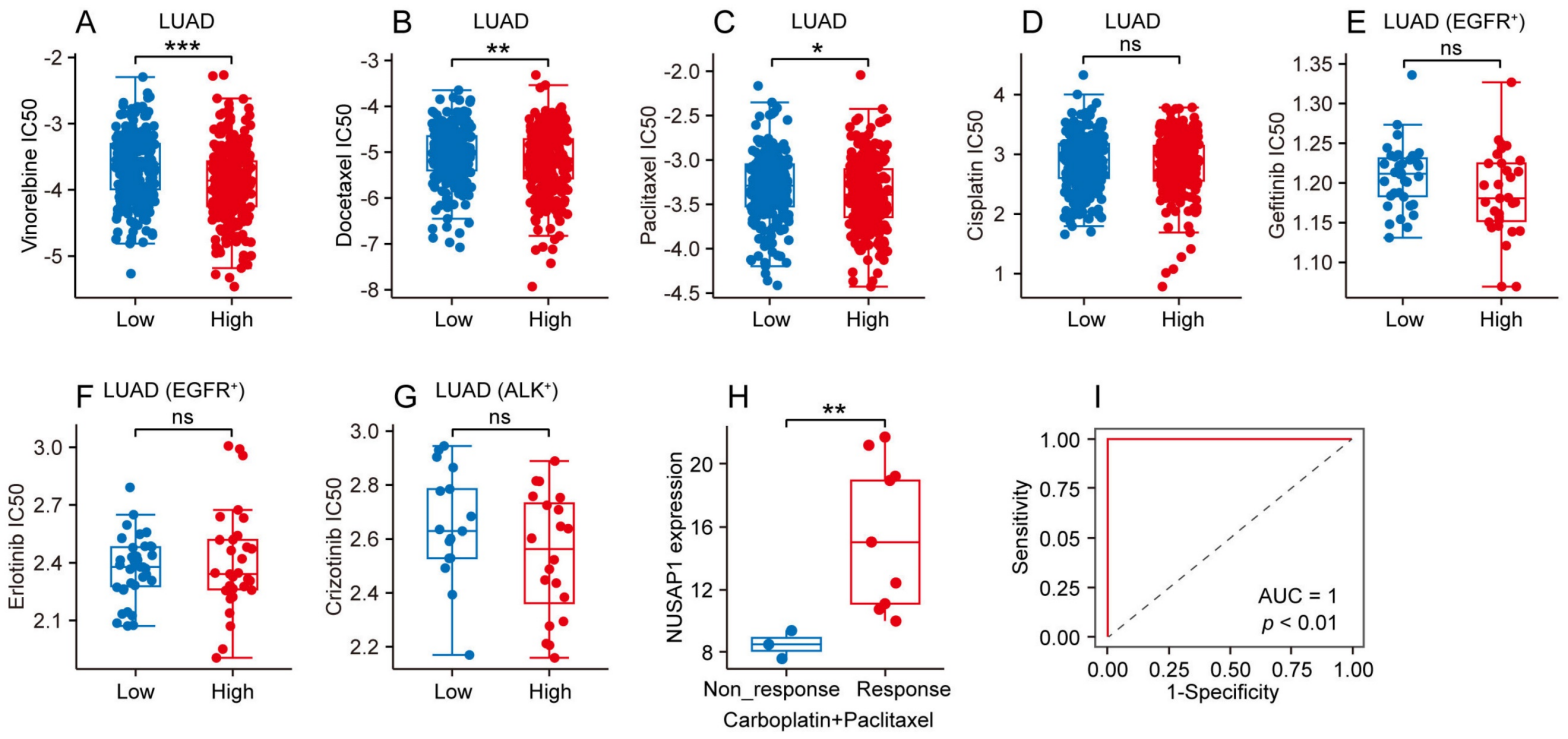
** Different chemotherapeutic and targeted therapeutic responses based on NUSAP1 expression in LUAD. (A-D)** Half maximal inhibitory concentration (IC50) of chemotherapeutic drugs including vinorelbine** (A)**, docetaxel **(B)**, paclitaxel **(C)** and cisplatin **(D)** in high- and low-NUSAP1 expression groups in LUAD. (^*^p<0.05, ^**^p<0.01, ^***^p<0.001, ns, not significant). **(E-F)** IC50 of targeted therapeutic drugs including gefitinib **(E)** and erlotinib **(F)** in high- and low-NUSAP1 expression groups in EGFR-mutant (EGFR^+^) LUAD. ns, not significant. **(G)** IC50 of targeted therapeutic drug crizotinib in high- and low-NUSAP1 expression groups in ALK-mutant (ALK^+^) LUAD. ns, not significant. **(H)** NUSAP1 expression in LUAD tissues with different response status to carboplatin + paclitaxel therapy. (^**^p<0.01). **(I)** The receiver operator characteristic (ROC) curve of NUSAP1 expression in predicting the response status of LUAD patients receiving carboplatin + paclitaxel therapy.

To further elucidate the predictive value of NUSAP1 in LUAD patients' response to conventional chemotherapy and targeted therapy, we analyzed data from GDSC database to compare the half maximal inhibitory concentration (IC50) of common chemotherapeutic and targeted therapeutic drugs between high- and low-NUSAP1 expression groups of LUAD patients. As depicted in **Figure 7 A-C**, the high-NUSAP1 expression group exhibited greater sensitivity to vinorelbine, docetaxel and paclitaxel as evidenced by lower IC50 compared to low-NUSAP1 expression group in LUAD. Conversely, no significant difference was found in the IC50 of cisplatin in LUAD, gefitinib and erlotinib in EGFR-mutated LUAD, or crizotinib in ALK-mutated LUAD between high- and low-NUSAP1 expression groups (**Figure 7 D-G**). Additionally, further analysis in CTR-DB database revealed that patients with LUAD who responded favorably to the combination therapy of carboplatin and paclitaxel exhibited higher NUSAP1 expression compared to non-responders (**Figure 7 H**). Notably, NUSAP1 expression was specific and sensitive in predicting the response status of LUAD patients receiving this combination therapy (**Figure 7 I**). These results suggest that high NUSAP1 expression in LUAD may serve as a predictor of a favorable response to conventional chemotherapy, thereby assisting physicians in providing personalized treatment plans for LUAD patients.

### NUSAP1 promotes LUAD cell proliferation

**Figure 8:**
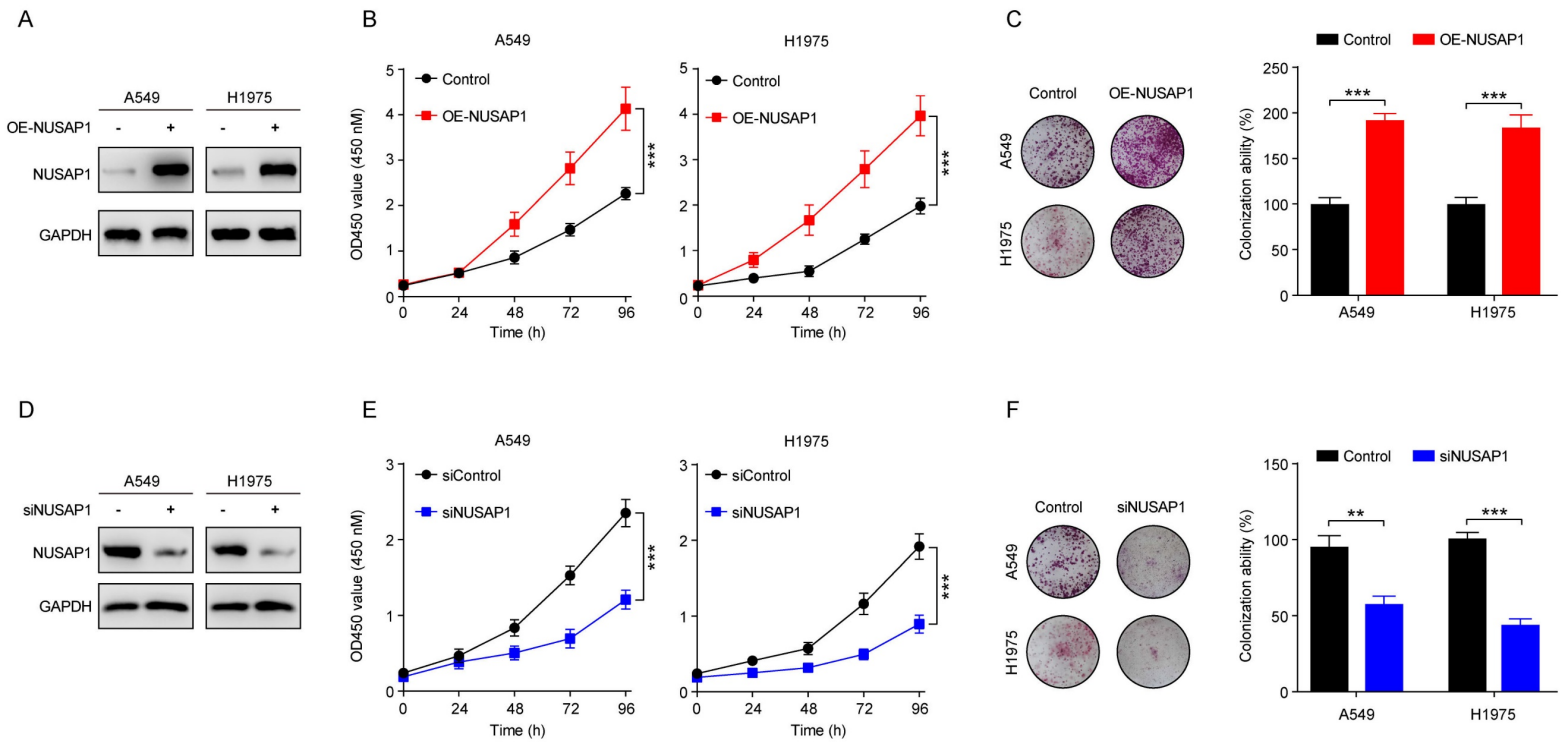
** NUSAP1 promotes LUAD cell proliferation. (A)** Cells were infected with overexpressing-NUSAP1 or negative control lentiviral particles, then selected by treatment with 2 μg/mL puromycin, and ultimately collected for detection of NUSAP1 expression by western blot assay.** (B)** Cells stably expressing either vector or NUSAP1 were subjected to CCK8 assay. Results are presented as means ± SD. (^***^p<0.001). **(C)** Cells stably expressing either vector or NUSAP1 were subjected to colony formation assay. The representative images (left) and quantitative results (right) were presented. Quantitative data are presented as means ± SD. (^***^p < 0.001). **(D)** Cells were transfected with siRNA-control or siRNA-NUSAP1 and collected 48 h later for detection of NUSAP1 expression by western blot assay. **(E)** Cells transfected with siRNA-control or siRNA-NUSAP1 were subjected to CCK8 assay. Results are presented as means ± SD. (^***^p < 0.001).** (F)** Cells transfected with siRNA-control or siRNA-NUSAP1 were subjected to colony formation assay. The representative images (left) and quantitative results (right) were presented. Quantitative data are presented as means ± SD. (^**^p<0.01, ^***^p<0.001).

Encouraged by these promising bioinformatic results, we next performed *in vitro* experiments to verify the impact of NUSAP1 on the proliferation of LUAD cells. To this end, we established LUAD cell lines (A549 and H1975) stably expressing NUSAP1 (**Figure 8 A**). Subsequent CCK8 assay results showed that NUSAP1 overexpression significantly promoted the proliferation of A549 and H1975 cells (**Figure 8 B**), which were corroborated by colony formation assay (**Figure 8 C**). On the contrary, we transiently transfected NUSAP1-siRNAs into A549 and H1975 cells to specifically knock down NUSAP1 expression (**Figure 8 D**). Subsequent CCK8 and colony formation assays showed that NUSPA1 knockdown significantly inhibited the proliferation of A549 and H1975 cells (**Figure 8 E-F**). These results suggest that NUSAP1 functions as an oncogene to promote cell proliferation of LUAD cells.

### NUSAP1 is upregulated by estradiol through ERβ activation

**Figure 9:**
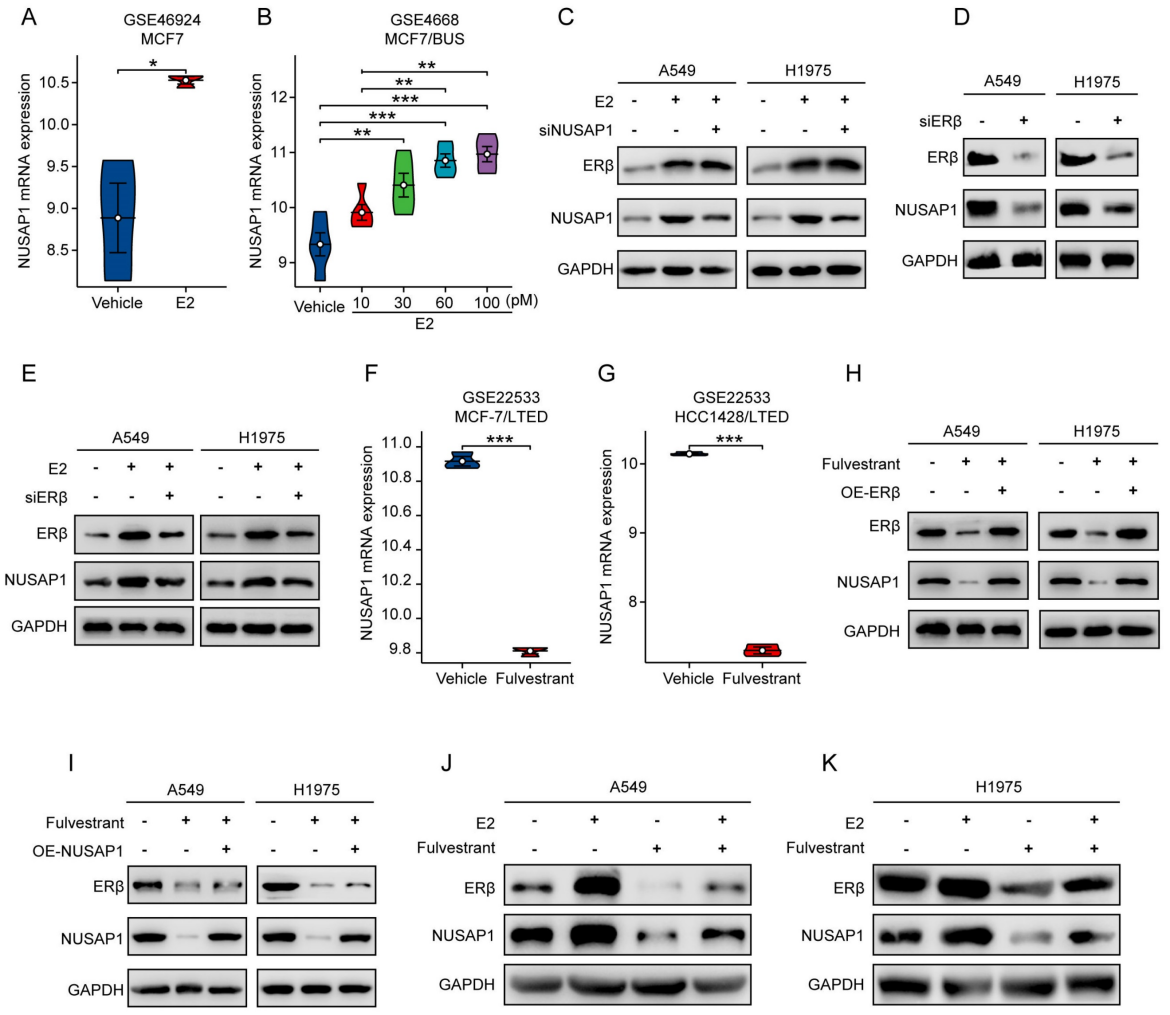
**NUSAP1 is upregulated by estradiol through ERβ activation. (A-B)** NUSAP1 expression between E2-treated breast cancer cell lines and the vehicle-treated control group in GSE46924** (A)** and GSE4668** (B)** datasets. (^*^p < 0.05, ^**^p<0.01, ^***^p < 0.001). **(C)** Cells were transfected with E2 alone (100 nM) or along with siRNA against NUSAP1 and collected 48 h later for detection of NUSAP1 and ERβ expression by western blot assay. **(D)** Cells were transfected with siRNA-control or siRNA-ERβ and collected 48 h later for detection of ERβ and NUSAP1 expression by western blot assay. **(E)** Cells were transfected with E2 alone (100 nM) or along with siRNA against ERβ and collected 48 h later for detection of ERβ and NUSAP1 expression by western blot assay. **(F-G)** NUSAP1 expression between E2-treated breast** (F)** or liver **(G)** cancer cell lines and the corresponding vehicle-treated control in GSE22533 dataset. (^***^p < 0.001). **(H)** Cells stably expressing either vector or ERβ were treated with fulvestrant (100 nM) and collected 48 h later for detection of ERβ and NUSAP1 expression by western blot assay. **(I)** Cells stably expressing either vector or NUSAP1 were treated with fulvestrant (100 nM) and collected 48 h later for detection of ERβ and NUSAP1 expression by western blot assay. **(J-K)** Cells were treated with E2 (100 nM) or fulvestrant (100 nM) alone or both combination for 48 h and then subjected to western blot assay for detection of ERβ and NUSAP1 expression.

Given the established oncogenic role of NUSAP1 in LUAD, we were interested in elucidating how NUSAP1 is regulated in LUAD. Interestingly, as aforementioned, we noted a significant enrichment in estrogen signaling pathway from the KEGG analysis of NUSAP1-related upregulated DEGs in TCGA-LUAD cohort (**Figure 5 H**). Since accumulating studies have consistently demonstrated the important role of estrogen in promoting lung carcinogenesis and development upon its receptor activation^28^, ^54^, ^55^, we speculated that estrogen might be involved in the regulation of NUSAP1 in LUAD. To test this hypothesis, first of all, we performed bioinformatics analysis of two GEO datasets comprising estradiol (E2)-treatment data. We found that NUSAP1 expression was significantly upregulated in E2-treated breast cancer cells compared to the vehicle-treated control in GSE46924 dataset (**Figure 9 A**). Consistently, analysis of GSE4668 dataset showed a concentration-dependent increase of NUSAP1 expression in breast cancer cells treated with increasing concentrations of E2 (**Figure 9 B**), indicating that E2 positively regulates NUSAP1 expression. Subsequently, we treated LUAD cell lines (A549 and H1975) with E2. As anticipated in **Figure 9 C**, E2 treatment activated ERβ as previous study reported^28^, accompanied with significant upregulation of NUSAP1. Moreover, E2-induced ERβ activation could not be reversed by NUSAP1 knockdown (**Figure 9 C**), while ERβ knockdown significant downregulated NUSAP1 expression (**Figure 9 D**) and reversed the upregulation of NUSAP1 induced by E2 (**Figure 9 E**), indicating that E2 promoted NUSAP1 expression via ERβ activation. It is well known that fulvestrant functions as a pure antiestrogen and a potent estrogen receptor (ER) antagonist that inhibits ERβ^56^, ^57^. Intriguingly, through analysis of GSE22533 dataset which comprises fulvestrant treatment data, we found that NUSAP1 was markedly downregulated upon fulvestrant treatment in both breast cancer and liver cancer cell lines (**Figure 9 F-G**). Consistently, we noted that blockade of ERβ activation by fulvestrant significantly suppressed NUSAP1 protein expression in both A549 and H1975 LUAD cells, which was reversed by ERβ overexpression (**Figure 9 H**), while NUSAP1 overexpression could not reverse fulvestrant-induced ERβ protein decrease (**Figure 9 I**). Furthermore, the co-treatment assay showed that fulvestrant sufficiently blocked the enhancement effect of E2 on ERβ/NUSAP1 axis (**Figure 9 J-K**).

Taken together, these results clearly suggest that NUSAP1 is upregulated by estradiol through ERβ activation.

### Fulvestrant suppresses LUAD growth through inhibiting ERβ/NUSAP1 axis *in vitro* and *in vivo*

**Figure 10:**
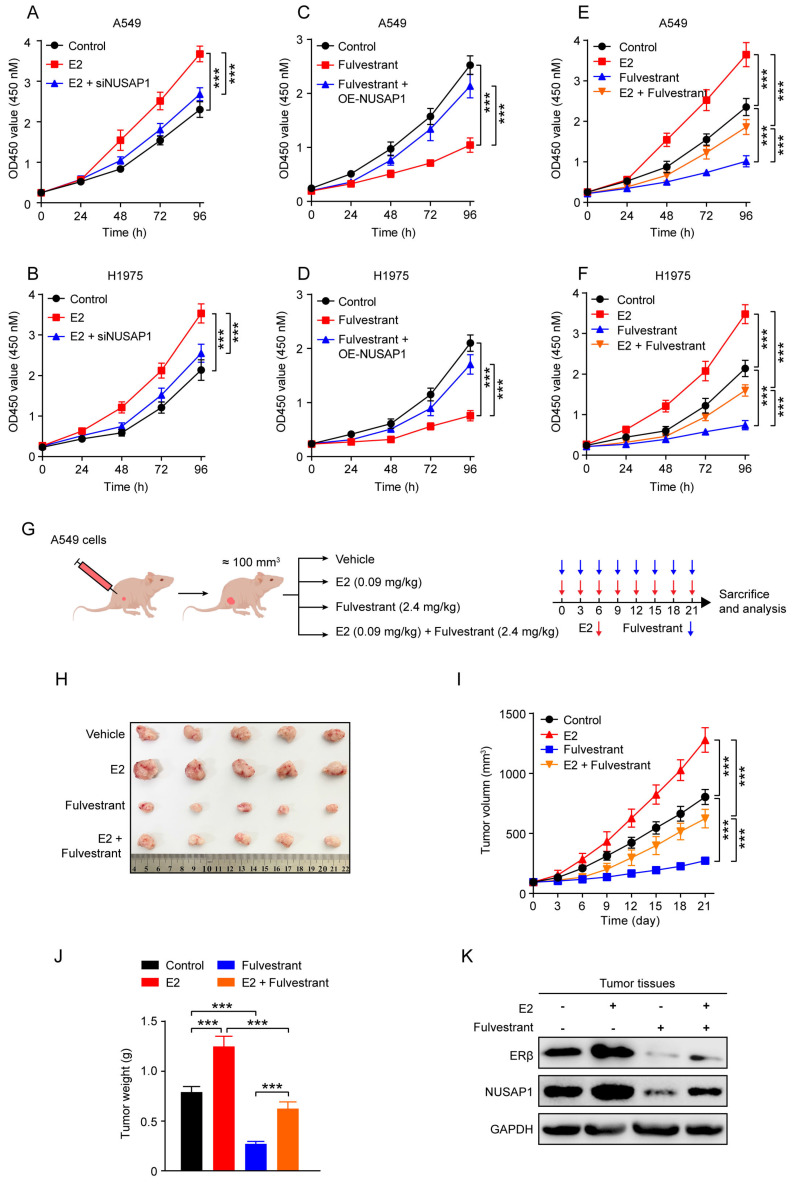
** Fulvestrant suppresses LUAD growth through inhibiting ERβ/NUSAP1 axis *in vitro* and* in vivo*. (A-B)** Cells were treated with E2 (100 nM) alone or along with siRNA against NUSAP1 for 48 h and then subjected to CCK8 assay. Results are presented as means ± SD. (^***^p < 0.001). **(C-D)** Cells stably expressing either vector or NUSAP1 were treated with fulvestrant (100 nM) and then subjected to CCK8 assay. Results are presented as means ± SD. (^***^p<0.001). **(E-F)** Cells were treated with E2 (100 nM) or fulvestrant (100 nM) alone or both combination for 48 h and then subjected to CCK8 assay. Results are presented as means ± SD. (^***^p < 0.001). **(G)** Schematic illustrating the *in vivo* experimental design. Ovariectomized (OVX) female nude mice were subcutaneously injected with A549 cells (5 × 10^6^). When the tumor volume reached about 100 mm^3^, the mice were randomly divided into four group and subcutaneously treated with vehicle (saline) or E2 (0.09 mg/kg, i.h, q3d) or fulvestrant (2.4 mg/kg, i.h, q3d) or E2 plus fulvestrant. After 21 days of drug administration, the mice were euthanized, and the tumors were harvested for analysis. **(H)** Images of the tumors.** (I)** The tumor growth curves. Results are presented as means ± SD. (^***^p < 0.001). **(J)** The tumor weight. Results are presented as means ± SD. (^***^p < 0.001). **(K)** The protein expression of NUSAP1 and ERβ in the tumor tissues detected by western blot assay.

The above results prompted us to examine whether blockade of ERβ/NUSAP1 axis by fulvestrant exhibits anti-LUAD effects. As expected, results from CCK8 assay showed that E2 significantly promoted the proliferation of A549 and H1975 cells, while NUSAP1 knockdown significantly attenuated this effect (**Figure 10 A-B**). On the contrary, fulvestrant significantly inhibited the proliferation of A549 and H1975 cells, which was significantly rescued by NUSAP1 overexpression (**Figure 10 C-D**).

Besides, we found that E2-induced LUAD cell proliferation could be sufficiently reversed by adding fulvestrant treatment (**Figure 10 E-F**). To corroborate these *in vitro* results, we established subcutaneous xenograft model by implanting A549 cells into the ovariectomized (OVX) female nude mice (**Figure 10 G**). The *in vivo* growth results showed that E2 significantly promoted tumor growth as evidenced by increased tumor volume and tumor weight, whereas fulvestrant treatment generated the opposite effects (**Figure 10 H-J**). Notably, the stimulatory effect of E2 on LUAD tumor growth was significantly reversed when co-treated with fulvestrant (**Figure 10 H-J**). Of important note, NUSAP1 protein expression in tumor tissues was significantly upregulated when ERβ was activated by E2, while such effect was significantly reversed by fulvestrant antagonising ERβ (**Figure 10 K**), which was consistent with the *in vitro* results as aforementioned (**Figure 9 J-K**). Taken together, these results suggest that fulvestrant suppresses LUAD growth through inhibiting ERβ/NUSAP1 axis *in vitro* and *in vivo*.

### Entinostat is a novel inhibitor of NUSAP1 and exhibits a significant growth inhibitory effect on LUAD growth *in vitro* and *in vivo*

**Figure 11:**
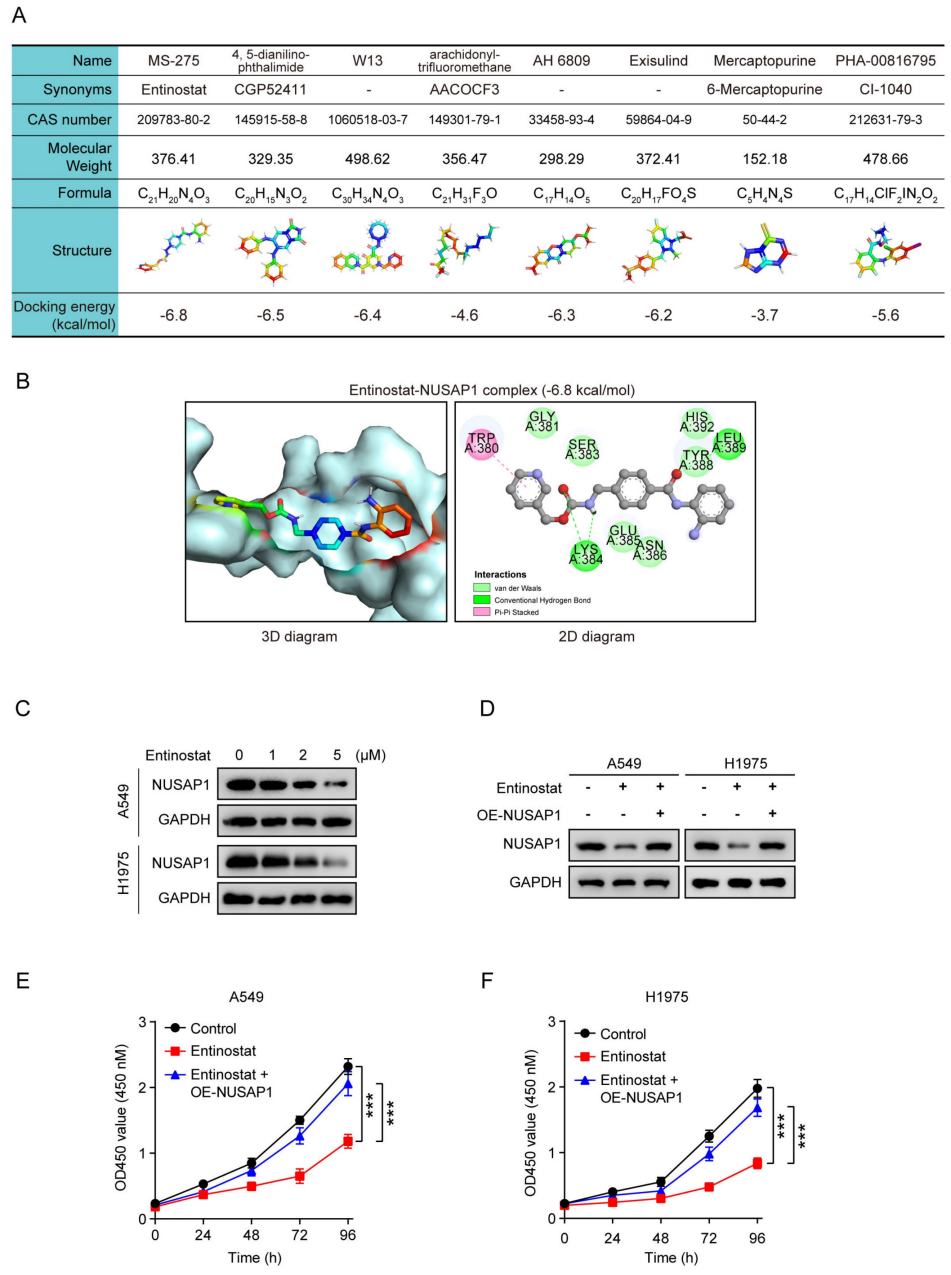
** Entinostat is a novel inhibitor of NUSAP1 and exhibits a significant growth inhibitory effect on LUAD proliferation. (A)** The information of eight compounds and docking energy of the eight compounds with NUSAP1 protein.** (B)** The 3D (left) and 2D (right) docking diagram of the interaction between entinostat and NUSAP1 protein. **(C)** Cells were treated with different concentrations of entinostat (0 μM, 1 μM, 2 μM, 5 μM) and collected 48 h later for detection of NUSAP1 expression by western blot assay.** (D)** Cells stably expressing either vector or NUSAP1 were treated with entinostat (5 μM) and collected 48 h later for detection of NUSAP1 expression by western blot assay. **(E-F)** Cells stably expressing either vector or NUSAP1 were treated with entinostat (5 μM) and then subjected to CCK8 assay. Results are presented as means ± SD. (^***^p < 0.001).

Finally, we wonder whether directly targeting NUSAP1 could also exert great therapeutic efficiency on LUAD. Due to no commercially available NUSAP1 inhibitors currently, we summarized eight potential small molecule compounds that may target NUSAP1 for the treatment of LUAD identified by the Connectivity Map^58^ analysis reported in a published literature^59^. We then further conducted molecular docking analysis to evaluate the affinity of NUSAP1 with the eight compounds. The details of the eight compounds and the docking results were listed in **Figure 11 A**, from which we found that the docking energy of entinostat-NUSAP1 complex (-6.8 kcal/mol) was lowest (**Figure 11 A, B**), indicating entinostat may have the strongest potential to inhibit NUSAP1, so we chose entinostat for further experimental verification. The western blot analysis showed that entinostat could inhibit the expression of NUSAP1 in A549 and H1975 cells in a dose-dependent manner (**Figure 11 C**), while NUSAP1 overexpression was able to reverse the entinostat-induced inhibitory effect on NUSAP1 (**Figure 11 D**). Moreover, CCK8 results showed that entinostat significantly suppressed the proliferation of both A549 and H1975 cells, which was significantly reversed by NUSAP1 overexpression (**Figure 11 E, F**), indicating that entinostat inhibited LUAD cell proliferation through targeting NUSAP1.

**Figure 12:**
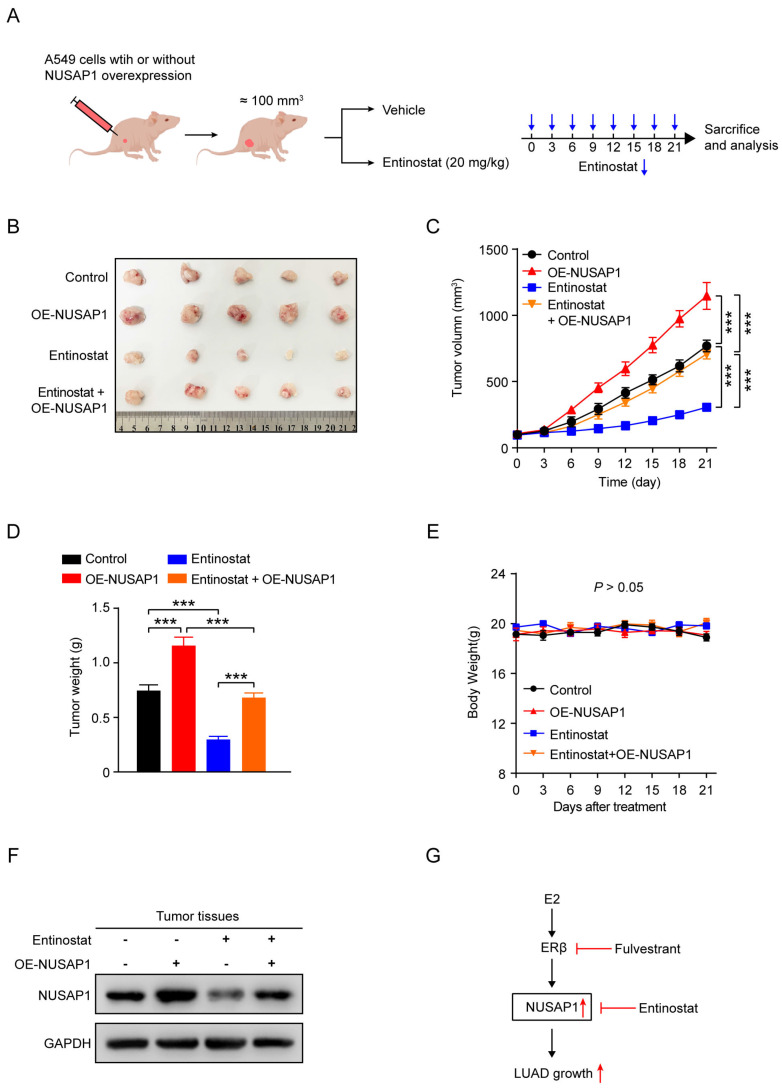
** Entinostat suppresses LUAD tumor growth via targeting NUSAP1 *in vivo*. (A)** Schematic illustrating the *in vivo* experimental design. A549 cells stably expressing either vector or NUSAP1 were subcutaneously injected into female nude mice. When the tumor volume reached about 100 mm^3^, the mice were intraperitoneally administrated with vehicle (saline containing 1% tween 80) or entinostat (20 mg/kg, q3d). After 21 days of drug administration, the mice were euthanized, and the tumors were harvested for analysis. **(B)** Images of the tumors. **(C)** The tumor growth curves. Results are presented as means ± SD. (^***^p < 0.001). **(D)** The tumor weight. Results are presented as means ± SD. (^***^p < 0.001). **(E)** The mouse body weight. Results are presented as means ± SD. **(F)** The protein expression of NUSAP1 and ERβ in the tumor tissues detected by western blot assay. **(G)** Schematic diagram showing the possible action mechanism by which E2, fulvestrant and entinostat act on the expression of NUSAP1 and LUAD growth.

To further verify the impact of entinostat *in vivo*, A549 cells stably expressing either vector or NUSAP1 were subcutaneously injected into nude mice to establish subcutaneous xenograft model, and then treated with entinostat (**Figure 12 A**). As shown in **Figure 12 B-D**, NUSAP1 overexpression was found to significantly trigger tumor growth as evidenced by increased tumor volume and tumor weight, while entinostat generated the significant inhibitory effects. Noteworthily, the entinostat-induced tumor growth inhibition was counteracted by NUSAP1 overexpression (**Figure 12 B-D**). In addition, no noticeable loss of appetite, nausea, vomiting, or significant weight loss was found in mice throughout the experiment. (**Figure 12 E**). Furthermore, consistent with *in vitro* results in **Figure 11 D**, western blot assays of tumors verified the significant inhibitory effects of entinostat on NUSAP1, which was significantly revered by NUSAP1 overexpression (**Figure 12 F**).

Taken together, these results suggest that entinostat is a potent inhibitor of NUSAP1 and exhibits a significant growth inhibitory effect on LUAD growth *in vitro* and *in vivo*, providing an optional therapeutic agent for the treatment of LUAD (**Figure 12G**).

## Discussion

Unperturbed mitosis is a pivotal biological process requiring intricate regulation mediated by diverse proteins. Among these, NUSAP1, a vital microtubule-binding and stabilizing protein, participates in regulating both early and late stages of mitosis^60^. Given the well-documented dysregulation of mitosis in various tumor types, the association between NUSAP1 and malignant tumor has been increasingly revealed. Aberrant expression of NUSAP1 has been observed in multiple types of cancers, and has been implicated in tumorigenesis and development, and associated with unfavorable clinical outcomes^9^, ^15^, ^61^, ^62^. In present study, we found elevated NUSAP1 expression in LUAD, which correlated with somatic mutations of multiple oncogenes including ALK, ROS1, RET and NTRK3 driver genes. In addition to a poor prognostic biomarker, we further found that NUSAP1 overexpression significantly promoted LUAD cell proliferation, while its knockdown exhibited the opposite effect, which was consistent with previous findings^20^, ^63^. These findings jointly revealed that NUSAP1 functions as an oncogene in LUAD. Conversely, contradictory results in cervical cancer showed that decreased NUSAP1 expression was correlated with poor prognosis^64^. We speculate that NUSAP1 may participate in discrepant functional process of mitosis in different types of cancer, resulting in opposing effects.

It is universally acknowledged that immune checkpoint inhibitors (ICIs) constitute an effective treatment strategy for LUAD. However, only a subset of patients benefits from ICIs. Although the combination of ICIs and chemotherapy enhances treatment efficacy in LUAD, the response rate remains approximately 50%^65^, ^66^. Therefore, it is of great importance to identify predictive biomarkers for selecting LUAD patients for ICIs and explore novel treatment strategies for LUAD.

Notably, our study revealed that LUAD patients with elevated NUSAP1 expression exhibited higher scores for TMB and NALs, which are commonly known predictors of the ICIs treatment efficacy 67-69. Furthermore, LUAD patients with high NUSAP1 expression displayed a greater proportion of immune cells such as CD8^+^ T cells, activated CD4^+^ memory T cells, M0 and M1 macrophages, as well as augmented expression of ICB-relevant genes, significantly contributing to the facilitation of anti-cancer immune activity. Based on our findings, it is plausible to postulate that high NUSAP1 expression may serve as a predictive biomarker to identify LUAD patients who may gain benefit from ICIs, which necessitates validation in clinical cohorts. Additionally, given that NUSAP1 may play a role in regulating the tumor microenvironment, modulating NUSAP1 expression may represent a promising therapeutic strategy to be combined with ICIs. Of note, we also found that higher NUSAP1 expression is associated with increased sensitivity to vinorelbine, docetaxel and paclitaxel, as well as the combined chemotherapy of carboplatin and paclitaxel. Such enhanced treatment response may signify an increased release of tumor neoantigens, which stimulates anti-cancer immune activity and potentially enhances the efficacy of ICIs^70^, ^71^. Therefore, for LUAD patients exhibiting higher NUSAP1 expression, combining ICIs and chemotherapy regimens that exhibit significant sensitivity and high response rates may be an efficient treatment strategy. However, the underlying mechanism governing the relationship between NUSAP1 and immune activity remains to be further elucidated.

In addition to being a potential predictive biomarker of benefit from ICIs for LUAD patients, NUSAP1 contributed to oncogenic function, indicating it is an effective therapeutic target for the treatment of LUAD. Clarifying how NUSAP1 is regulated has enormous clinical translational significance for developing effective therapeutic strategy. Notably, our study showed that the estrogen signaling pathway was enriched in NUSAP1-related upregulated DEGs, and E2 was proved as a key cause for NUSAP1 upregulation in LUAD. Over the past decade, gender differences in NSCLC have been increasingly recognized. Notably, the incidence of lung cancer, particularly LUAD, has escalated among women^72^, ^73^. Additionally, pre-menopausal women tend to be diagnosed with more advanced stages of the disease, exhibiting a poorer prognosis compared to their post-menopausal counterparts and male patients^74^-^76^. Conversely, hormone replacement therapy has been associated with an accelerated progression of lung cancer, in particular NSCLC^77^. These findings indicate that sex hormones play an influential role in the progression of NSCLC, underscoring their significance. Accumulating evidence has highlighted estrogen and its related pathways as potential contributors to lung cancer development^26^-^28^, yet the underlying mechanisms remain incompletely elucidated. In this study, we confirmed that E2 significantly promoted LUAD progression *in vitro* and *in vivo*. Additionally, our results revealed that NUSAP1 was significantly upregulated by E2 via ERβ activation, thereby promoting LUAD growth, which has enhanced our understanding about the oncogenic role of estrogen and the upstream regulatory mechanisms of NUSAP1 in LUAD. Consequently, blocking the E2/ERβ/NUSAP1 axis may serve as a promising treatment strategy in LUAD.

Previous studies have shown that fulvestrant, a pure antiestrogen and a potent estrogen receptor (ER) antagonist, exhibited a distinct anti-cancer effect on LUAD^78^, ^79^, yet the precise mechanism underlying this effect remains elusive. In our current study, we have validated the inhibitory effect of fulvestrant on the proliferation of LUAD cells. Besides, we also uncovered that fulvestrant suppressed the expression of NUSAP1 via inactivating ERβ, subsequently impeding the progression of LUAD. Our finding further corroborates the involvement of the E2/ERβ/NUSAP1 axis in LUAD, indicating that targeting estrogen receptor signaling through fulvestrant presents a promising therapeutic strategy for LUAD. However, it is crucial to acknowledge that estrogen-estrogen receptor signaling pathway also plays a pivotal role in regulating numerous tissue and organ functions during physiological processes^21^-^23^. Therefore, directly blocking NUSAP1 instead of applying fulvestrant may emerge as an optimal therapeutic option for the treatment of LUAD.

Currently, there is no FDA-approved NUSAP1 inhibitor. A previous study performed Connectivity Map^58^ analysis and demonstrated several small molecular compounds with potential ability to inhibit NUSAP1^59^, necessitating further validation in preclinical study. Among these previously reported compounds, we further confirmed that entinostat is a novel and promising inhibitor of NUSAP1. Entinostat, previously identified as a class I selective histone deacetylase (HDAC) inhibitor, has found to exert inhibitory effects on cell proliferation and promote apoptosis in breast cancer^80^. In clinical trial, entinostat has displayed anticancer effects as evidenced by improved progression-free survival when combined with exemestane compared to exemestane alone^81^. Solta and his colleagues demonstrated that entinostat significantly enhances the efficacy of chemotherapy in small cell lung cancer by inducing S-phase arrest and reducing base excision repair^82^. Intriguingly, a recent study reported that entinostat promotes inflammatory remodeling of the tumor microenvironment, contributing to an enhancement of epitope spreading and antitumor immunity^83^. In current study, we demonstrated that entinostat significantly suppressed NUSAP1 expression and effectively halted LUAD growth in both preclinical *in vitro* and *in vivo* models. Conversely, NUSAP1 overexpression reversed these inhibitory effects. However, given that epigenetic aberrations are pivotal in lung carcinogenesis, we cannot discount the potential role of entinostat as a HDAC inhibitor in modulating histone acetylation status, a key factor in cancer development. Nevertheless, our findings provide compelling evidence that the anti-cancer effect of entinostat is, at least partially, mediated through the inhibition of NUSAP1 expression. In addition, no obvious adverse event was found with entinostat treatment. However, whether entinostat regulates NUSAP1 expression also associated with its modulation on histone acetylation are yet to be fully elucidated. In addition, the therapeutic role of entinostat in LUAD remains to be further validated in clinical trials. Future studies focused on addressing these issues would be necessary.

## Conclusion

In conclusion, NUSAP1 is elevated in LUAD and contributes to a poor overall survival. NUSAP1 is upregulated by estradiol through ERβ activation, thereby promoting LUAD growth. Entinostat was identified as a novel inhibitor of NUSAP1, exhibiting potent anti-LUAD growth effects. Pharmacological targeting ERβ/NUSAP1 axis with fulvestrant (ERβ antagonist) or entinostat (novel NUSAP1 inhibitor) may represent effective alternative therapeutic strategies for patients with LUAD.
